# Prognostic Nomogram for Sorafenib Benefit in Hepatitis B Virus-Related Hepatocellular Carcinoma After Partial Hepatectomy

**DOI:** 10.3389/fonc.2020.605057

**Published:** 2021-02-11

**Authors:** Wei Dong, Kai Yan, Hua Yu, Lei Huo, Zhihong Xian, Yanqing Zhao, Jutang Li, Yuchan Zhang, Zhenying Cao, Yong Fu, Wenming Cong, Hui Dong

**Affiliations:** ^1^ Department of Pathology, Eastern Hepatobiliary Surgery Hospital, the Second Military Medical University, Shanghai, China; ^2^ Key Laboratory of Signaling Regulation and Targeting Therapy of Liver Cancer, the Second Military Medical University, Shanghai, China; ^3^ The Fifth Department of Hepatic Surgery, Eastern Hepatobiliary Surgery Hospital, the Second Military Medical University, Shanghai, China; ^4^ Department of Radiology, Eastern Hepatobiliary Surgery Hospital, the Second Military Medical University, Shanghai, China; ^5^ Department of Gynaecology and Obstetrics, Tong Ren Hospital, Shanghai Jiao Tong University of Medicine, Shanghai, China

**Keywords:** hepatocellular carcinoma, sorafenib, personalized therapy, hepatitis B virus, nomogram, partial hepatectomy, prognosis

## Abstract

**Background:**

Predicting the long-term prognosis of individuals who experienced sorafenib treatment following partial hepatectomy due to hepatitis B virus (HBV) related hepatocellular carcinoma (HCC) is difficult. This work aims to create an effective prognostic nomogram for HBV related HCC patients who are receiving sorafenib treatment as adjuvant therapy after surgery.

**Methods:**

A total of 233 HBV-related HCC patients treated with or without sorafenib following partial hepatectomy at the Eastern Hepatobiliary Surgery Hospital from 2008 to 2013 were matched with propensity score matching analysis. The optimal cut-off point of the overall survival (OS) factor level was determined by x-tile. The selection of indicators was based on clinical findings. The Cox regression model with an interaction term was employed for evaluating the predictive value. Using a multivariate Cox proportional hazards model, a nomogram was subsequently formulated to analyze 111 patients treated with sorafenib. The nomogram’s discriminative ability and predictive accuracy were determined using the concordance index (C-index), calibration, and ROC curve.

**Results:**

The matched sorafenib cohort of 111 patients and control cohort of 118 patients were analyzed. Subgroup analysis revealed that low GPC3, pERK, pAKT, serum AFP levels, without MVI, under 50 years old, male, TNM stage I/II and BCLC stage 0/A were significantly associated with a better OS in patients subjected to sorafenib treatment compared to those without sorafenib treatment after surgery. Multivariate analysis of the sorafenib cohort revealed GPC3, pERK, pAKT, serum AST, and BCLC stage as independent factors for OS, and all were included in the nomogram. The survival probability based on the calibration curve showed that the prediction of the nomogram was in good agreement with the actual observation. The C-index of the nomogram for predicting survival was 0.73(95% CI, 0.67–0.78). The area under the ROC curve (AUC) for the nomogram to predict the survival for 1, 3, and 5-year was 0.726, 0.816, and 0.823, respectively.

**Conclusion:**

This proposed nomogram shows the potential to make a precise prediction regarding the prognosis of HBV-related HCC patients and may help to stratify patients for personalized therapy following partial hepatectomy.

## Background

Hepatocellular carcinoma (HCC) is the predominant primary liver cancer. Liver cancers are the 4^th^ leading cause of cancer-related deaths and the 6^th^ major cause of morbidities around the globe. The World Health Organization (WHO), based on its annual projections, estimates that over a million people will die of liver cancer in 2030 (1). Several randomized studies that tested adjuvant treatments, such as chemotherapy, interferon (IFN), internal radiation, chemoembolization, retinoids, and immune therapies, have not yet proven beneficial or lead to uncertain outcomes, hence are not recommended for clinical practice (2, 3). In the past decade, the treatment of advanced HCC has improved significantly (4).

Sorafenib is the first approved drug for the systemic therapy of advanced-stage HCC. All other therapies have only recently exhibited clinical efficacy. The United States Food and Drug Administration (US FDA) has approved Lenvatinib, as first (5) or regorafenib (6), nivolumab (7), cabozantinib (8), and ramucirumab as second-line treatments. Being a multi-kinase inhibitor, sorafenib targets the mitogen-activated protein kinase/extracellular signal-regulated kinase (MAPK/ERK) pathway, the vascular endothelial growth factor receptors-1/2/3 (VEGFR1/2/3), and c-KIT, among other targets, that provide a median survival advantage of nearly 3 months and reduce the risk of mortality by 31% in patients with an advanced stage of HCC (4). The STORM trial is the first randomized trial to evaluate the anti-recurrence effect of systemic therapy after liver resection or ablation. But the results failed to support the effectiveness of adjuvant sorafenib. However, this study excluded patients with AFP concentration more than 400 ng/ml, tumor size smaller than 2 cm, and macrovascular invaded HCC (9). The subsequent BIOSTORM study showed that 30% of patients with a specific genetic signature may benefit from sorafenib (4). A clinical trial showed reduced mortality and prolonged overall survival for adjuvant sorafenib in HCC patients after curative resection (10). A retrospective study showed that using sorafenib as adjuvant therapy after liver resection for HCC significantly reduced the recurrence in the sorafenib arm compared to that in the control arm (11). Sorafenib acts by VEGF signaling driven angiogenesis as well as cell proliferation mediated by MAPK/ERK (12). Besides, sorafenib is known to impact both endothelial as well as tumor cells (12). Although numerous investigations have been brought forward, the reliable predictive biomarkers (including targets of sorafenib such as VEGF or MAPK/ERK) of sorafenib responses have not been ascertained so far in HCC patients. Although sorafenib is beneficial in some patient subgroups, the latest meta-analysis of individual data from Asia-Pacific HCC and phase 3 SHARP trials revealed that sorafenib has significantly greater benefits for patients with HCV etiology and exclusive liver disease (13, 14). In terms of biomarkers, the correlation analysis of the SHARP trial showed that sorafenib treatment had a non-significant trend to improve survival in tumors with low plasma concentration of hepatocyte growth factor (HGF) and high c-Kit (15). Several efforts have been made for screening biomarkers, and predicting the responses of sorafenib as well as the prognosis of patients. Nevertheless, no single biomarker has been identified to predict sorafenib efficacy to date. In this scenario, it is highly important to investigate the association between clinicopathological index or biomarkers and sorafenib advantage in HCC. This needs a characteristic prognostic predictive model for the selection of patients to improve therapeutic efficacy.

Glypican-3 (GPC3) is a glycoprotein of oncofetal type and is found anchored to the cellular membrane through the glycophosphatidylinositol anchor. In an adult healthy liver, no GPC3 expression is obvious. In contrast, GPC3 is overexpressed in HCC. The GPC3 protein and gene expression in serum and tumor tissues of HCCs were higher compared to non-malignant healthy livers (16, 17). The prognostic value of serum GPC3 level and tumor cell GPC3 immunoreactivity as a biomarker has already been well established in patients with HCC. In addition, being a novel target molecule for therapeutic agents, GPC3 has also attracted much attention, and its clinical trials are in progress (18). So far, there is no investigation for exploring the association between the GPC3 expression and the prognosis of HCC patients subjected to sorafenib treatment. In liver cancer cells, MAPK/ERK and PI3K/Akt are two of the major prooncogenic signaling pathways. These two signaling pathways are often hyperactivated and dysregulated in HCC and have a regulatory role in survival, cell differentiation, and proliferation (19–25). Triggering of signaling pathways of MAPK and PI3K-AKT-mTOR had a poor outcome, and pERK (phosphorylated extracellular signal-regulated kinase) and pAKT (phosphorylation of protein kinase B) are the most common surrogate of AKT and MAPK pathway activation (4). Serum AST is an independent risk factor predicting prognosis has been included in many HCC prediction systems (26, 27). Previous investigations involving HCC patients subjected to sorafenib treatment have focused on an advanced stage of the disease; however, this study also included the early and very early stage of HCC within the BCLC staging system(the very early stage of HCC was single nodule; early stage of HCC was 2–3 nodules, all ≤3 cm) (1). In this study, we screened the sorafenib biomarker and clinicopathological index in HCC patients related to the hepatitis B virus and treated with sorafenib following partial hepatectomy. We then used a multivariate Cox proportional hazards model to establish a nomogram to carry out an analysis of the 111 patients treated with sorafenib.

For most cancer types, nomograms have been developed (28–30). The use of nomograms is more beneficial than traditional staging systems, therefore nomograms are often put forth as an alternative or as entirely new standards (31–34). To our knowledge, this is the first attempt to construct an effective prognostic nomogram for hepatitis B virus-related HCC patients treated with sorafenib after partial hepatectomy.

## Methods

### Patients and Treatment

**Figure 1:**
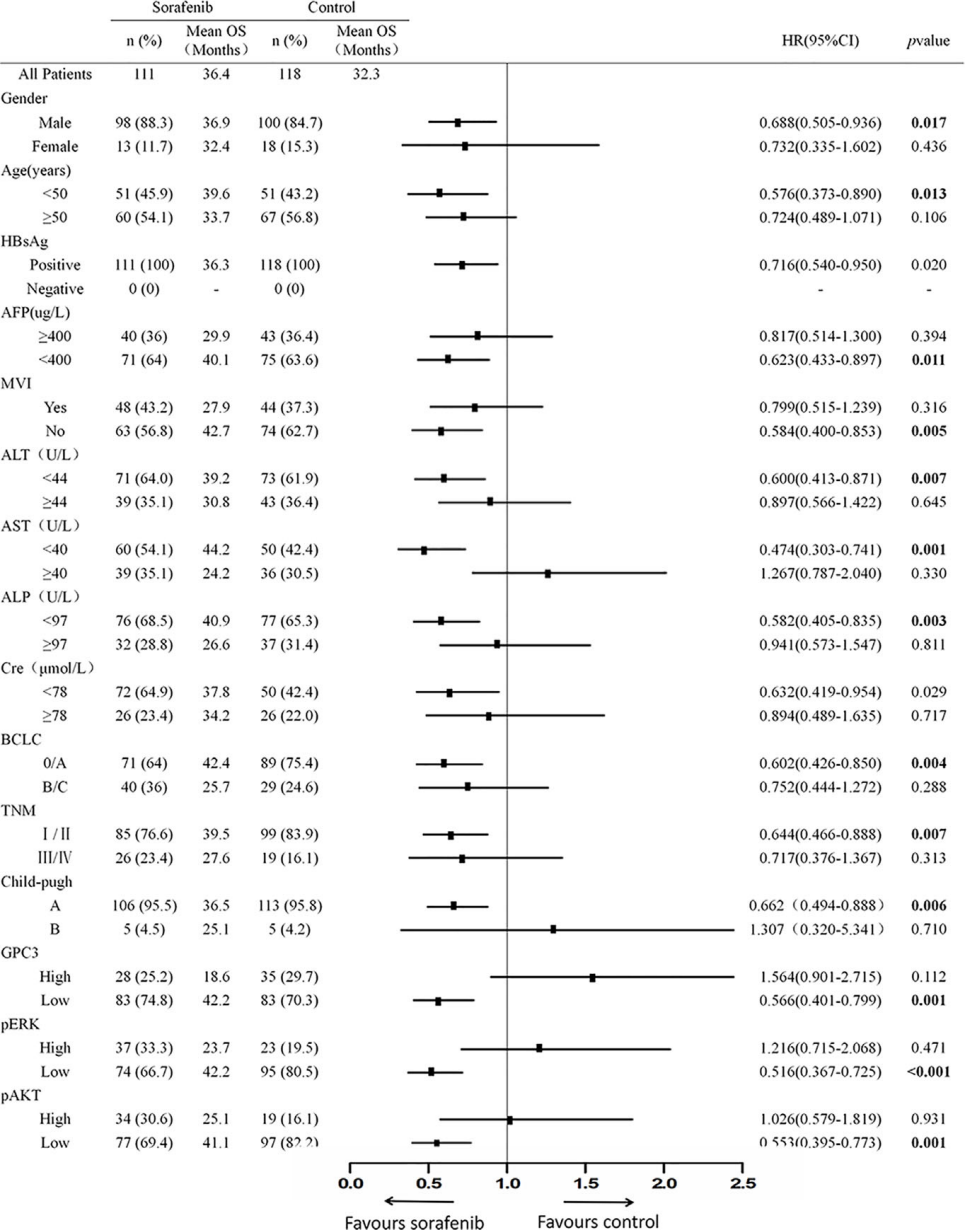
The Baseline Characteristics of Subgroup. The p value of interactions between treatment and biomark levels or clinical variables were also shown.

To investigate different aspects related to the advantages of sorafenib in HCC patients, 233 patients who went through surgery between April 2008 and February 2013 at Eastern Hepatobiliary Surgery Hospital (EHBH) were enrolled in this study. Propensity analysis was conducted to balance the bias in the relevant clinical characteristics of patients between the sorafenib and control groups and to identify patients with similar baseline characteristics, therefore, imitating a randomized controlled trial. Thus, all the patients in the sorafenib and control groups were subjected to propensity score matching analysis based on the baseline characteristics of patients of HCC. Based on the propensity score matching analysis, 118 out of 120 HCC patients without sorafenib therapy were matched to 111 out of 113 HCC patients who have received sorafenib therapy by propensity scores. Of them, 111 patients had been given sorafenib therapy following partial hepatectomy and were referred to as the sorafenib group. The 118 patients who did not receive sorafenib were referred to as the control group (**Table S2**).The inclusion criteria were included as (1) preoperative liver function was Child-Pugh A/B and the diagnosis was HCC; (2) received sorafenib treatment within a month following surgery and continued until death or more than one year. Sorafenib was administered orally at a dose of 400 mg twice daily. The drug was discontinued when more significant toxicity occurred; (3) hepatitis B core antibody (HBcAb) and/or hepatitis B surface antigen (HBsAg) were positive whereas hepatitis C antibody was negative; (4) the HCC patients had not been exposed to any pre-surgical treatment such as chemoembolization, high-intensity focused ultrasound or radiofrequency ablation. Their detailed clinicopathological features are depicted in **Figure 1**. Slides from each surgically resected tissue were prepared using hematoxylin and eosin (H&E)-stained and were examined by two accomplished hematopathologists (HD and W-MC). The primary HCC specimens were collected from patients for microarrays construction and immunohistochemistry (IHC) staining. **Figure S1** shows the flow diagrams of patients.

### Surgical Procedure

All surgeries were performed by the surgical team following standard techniques. Surgery was performed through a bilateral subcostal incision. The abdominal cavity was carefully searched for the extent of local disease and extrahepatic metastases. Intraoperative ultrasound was performed to assess the number and the size of the lesions, and to assess the relationship of the tumor to vascular structures. Pringle’s maneuver was applied to occlude the blood inflow of the liver with cycles of 15 min clamp time and 5 min unclamped time. Liver resection was carried out by a clamp-crushing method (35).

### Follow-Up

During the first-year after surgery, patients were followed up once every 2–3 months. After one year, they were followed up every 3–6 months. Assessments of liver function and serum tumor markers were carried out at every follow-up. Tumor recurrence was suspected on detection of new hepatic lesions on ultrasound or by a progressive and continuous elevation of serum AFP (>100 ng/ml). The serum AFP levels of these patients were also regularly monitored to check whether the AFP level of the patient had fallen to normal after the operation, or the patient had a normal AFP level before the operation. The diagnosis of recurrence was confirmed by dynamic CT scan or MRI. Further investigations (such as chest CT, full-body bone scan, and positron emission tomography-CT) were performed to confirm extrahepatic metastases (35). Overall survival (OS) and time to recurrence (TTR) were used as primary end points. The time duration between surgery and last follow-up exam or the death was termed as OS. Calculation of TTR was carried out from the date of surgery to the diagnosis of metastasis or recurrence.

### Tissue Microarrays, Immunohistochemistry, and Scoring

A total of 229 specimens were selected, and the representative core of each specimen was utilized to construct tissue microarrays. IHC was carried out and samples were measured according to previous reports (36, 37). The system for imaging included a CCD camera by Leica, DFC420, linked to a Leica DM IRE2 microscope obtained from Leica Microsystems Imaging Solutions, Cambridge, UK. The representative field images were taken from individual core under 200×magnification employing Leica QW in Plus v3 software. Counting and measurement of the photographs IOD were carried out with software of Image-Pro plus V6.0 (Media Cybernetics, Bethesda, MD, USA), and the parameters used were IOD and Area sum. Dilution of the Primary antibodies was done as follows: a rabbit monoclonal [SP86] to Glypican 3 (ab95363; Abcam, Hong Kong; 1:100 dilution, cytomembrane staining), a monoclonal rabbit antibody against Erk1/2 (137F5)(4695; Cell Signaling Technology, Danvers, MA, USA; 1:100 dilution, cytoplasmic staining), a rabbit monoclonal antibody against AKT1 (D9R8K)(75692, Cell Signaling Technology, Danvers, MA, USA; 1:200 dilution, cytoplasmic staining).

### Statistical Analysis

Identification of risk factors was accomplished *via* statistical analyses carried out with SPSS V22.0 software (IBM, Chicago, IL). Grouping of the categorical variables was completed according to the clinical findings, and the decisions for groups were made before modeling. A comparison of the continuous variables was made using the Mann–Whitney test for variables with the abnormal distribution. The sample size was calculated using the PASS 15 software. The HCC patients treated with or without sorafenib after partial hepatectomy were matched with propensity score matching analysis, using MatchIt4.0.0 packages in R version 4.0.1 (http://www.r-project.org/). The optimal cut-off points for the OS were calculated using the X-Tile statistical package (version 3.6.1, Yale University, New Haven, CT, USA). X-tile plot shows the presence of significant HCC subpopulations, and a two-dimensional projection of each possible subpopulation was used to show the robustness of the relationship between an outcome and a biomarker (38). The extent of quantitative factors such as, -AST, serum ALT -ALP, -Cre, GPC3, pERK, pAKT were assessed by creating X-tile plots. Kaplan–Meier method was employed to draw the survival curve while the log-rank test was used for their comparison. Multivariate analyses were carried out employing the Cox regression analysis. The estimated values were used for time-dependent ROC (receiver operating characteristic) analysis. A nomogram was created based entirely on the outcomes of multivariate analysis, using rmS6.0-0 packages in R version 4.0.1(http://www.r-project.org/). Finally, using the backward step-down selection process bath on the Akaike information criterion, a model selection process was implemented (39). Concordance index (C-index) was employed to estimate the performance of the Nomogram and further evaluated by comparing the nomograms predictions versus the Kaplan–Meier method-based survival probabilities. These activities were initiated using Bootstraps with 1,000 resample. The accuracy of the prognostic prediction increased with an increase in the value of C-index (26). *P* values less than 0.05 were considered statistically significant.

## Results

### Clinicopathologic Characteristics of Patients

Sample size calculation showed that 216 patients needed to be randomized, and the power value was 0.8012 (**Table.S1**). 118 of 120 HCC patients without sorafenib therapy were matched to 111of 113 HCC patients who have received sorafenib therapy by propensity scores. The clinicopathologic characteristics of patients in the sorafenib and control cohorts are listed in **Figure 1**. For the sorafenib cohort, the mean follow-up time was found to be 48.8 months, (ranging from 12.8 to 126.5 months), the mean TTR came out to be 14.4 months (ranging from 1.3 to 98.7 months) whereas the mean OS was 36.4months (ranging from0.9 to 119.6 months). The mean follow-up time in the control cohort was 47.1 months (range, 4.9–111.5 months) whereas the mean TTR and mean OS was 11.6 months (range, 1.0 to 110.1 months) and 31.9months (range, 3.1 to 111.5 months) respectively.

### The Association Between Indicators and Sorafenib Benefit in Hepatocellular Carcinoma Patients

**Figure 2:**
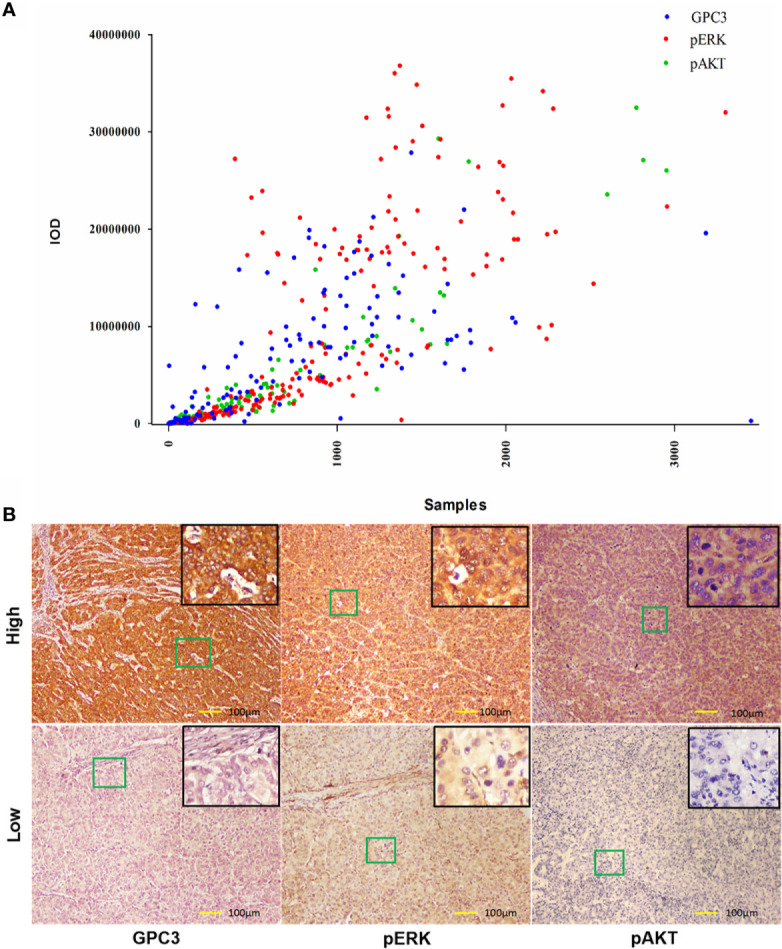
Expression of GPC3, pERK and pAKT in Hepatitis B Virus related HCC. **(A)** Immunohistochemical expression of GPC3, pERK and pAKT in HBV-related HCC. A scatter plot of samples and IOD for each marker was obtained. **(B)** Representative images of IHC staining of GPC3, pERK and pAKT from indicated patients were shown. Scale bar=100 µm.

To investigate the clinicopathologic characteristics in HCC patients and biomarker expression in HCC specimens and their relationship with the outcome of the patient, pathologic features, serological indicator, HCC staging systems, and biomarkers were selected based on the clinical findings. IHC staining was employed for the detection of the expression of biomarkers including GPC3, pERK, and pAKT in postoperative HCC specimens of 229 patients, followed by quantification and scoring. **Figure 2** shows the representative images of expressed biomarkers in HCC specimens. Further, to define the optimal cut-off points of those biomarkers and serological indicator levels, X-Tile was used for traversing expression of the biomarkers and serological indicator value as the cutoff point for dividing the patients and estimating the magnitude sorafenib benefits against control in the high- or low-level groups. Patients were also grouped according to the pathologic features and HCC staging systems. According to sorafenib treatment status, subgroup analysis revealed that low levels of GPC3 (cut-off value of IOD value was 57.1 × 10^5^, p = 0.001), pERK (cut-off value of IOD value was 16.8 × 10^6^,p < 0.001) and pAKT (cut-off value of IOD value was 18.3 × 10^5^, p = 0.001) were related to better OS, and survival advantages of sorafenib treatment have also been witnessed in HCC in male patients (*p* = 0.017), Age <50 years (*p* = 0.013), the lack of MVI (*p* = 0.005), AFP <400 μg/L (*p* = 0.011), ALT <44 U/L (*p* = 0.007), ALP <97 U/L (*p* = 0.003), AST <40 U/L (*p* = 0.001), BCLC stage 0/A (*p* = 0.004), TNM stage I/II (*p* = 0.007) or Child-Pugh stage A (*p* = 0.006). However, in a high level of GPC3, pERK, pAKT, female, age >50 years, positive MVI, AFP≥400 μg/L, ALT ≥44 U/L, ALP ≥97 U/L, AST ≥40 U/L, BCLC stage B/C, TNM stage III/IVor Child-Pugh stage B patients, the benefit of sorafenib was not statistically significant (**Figure 1**). The Kaplan–Meier analysis of OS also gave consistent results (**Figure S2**). Besides, a significant interaction was also detected between the treatment and these factors (**Table 1**).

**Table 1:** | Univariate and multivariate analysis of OS of patients receiving adjuvant sorafenib.

	Univariate analysis		Multivariate analysis	
Variables	HR (95% CI)	*p* value	HR	*p* value
Gender (Female vs Male)	0.952(0.476-1.904)	0.890		
Age (year)	1.000(0.982-1.017)	0.960		
MVI (Yes vs No)	0.580(0.378-0.890)	**0.013**	–	0.826
Tumor number (Single or Multiple)	1.176(0.959-1.443)	0.119		
AFP (μg/L)	1.000(1.000-1.001)	0.182		
Child-puch (A vs B)	1.956(0.897-4.264)	0.091		
ALT (U/L)	1.001(1.000-1.002)	0.091		
AST (U/L)	1.001(1.000-1.002)	**0.044**	1.001(1.000-1.002)	**0.043**
ALP (U/L)	1.003(1.001-1.005)	**0.013**	–	0.096
Cre (μmol/L)	0.988(0.969-1.007)	0.207		
BCLC (0/A vs B/C)	2.245(1.447-3.483)	**<0.001**	1.690(1.012-2.824)	**0.045**
TNM (I/II vs III/IV)	1.767(1.103-2.831)	**0.018**	–	0.871
GPC3 (High vs Low)	0.333(0.207-0.535)	**<0.001**	0.384(0.218-0.677)	**0.001**
pAKT (High vs Low)	0.436(0.277-0.685)	**<0.001**	0.514(0.308-0.857)	**0.011**
pERK (High vs Low)	0.398(0.258-0.615)	**<0.001**	0.582(0.341-0.994)	**0.048**

HBsAg, hepatitis B virus surface antigen; AFP, α-fetoprotein; MVI, macroscopic vascular invasion; BCLC, Barcelona Clinic Liver Cancer Staging; TNM, Tumor Nodes Metastasis; statistically significant values are bold.

### Independent Prognostic Factors in the Sorafenib Cohort

**Figure 3:**
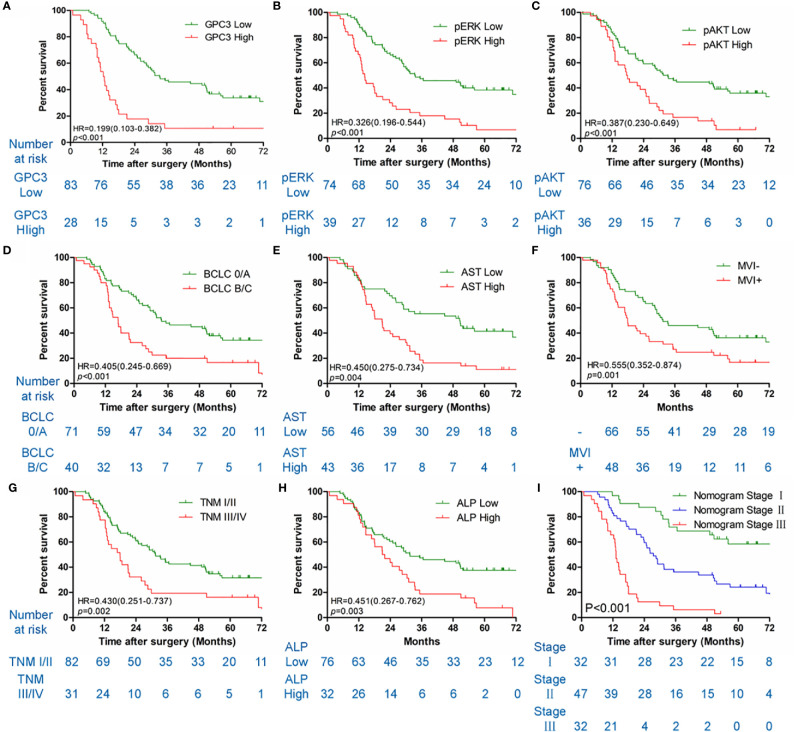
Kaplan-Meier survival curves of OS in the sorafenib cohort. GPC3 **(A)**, pERK **(B)**, pAKT **(C)**, BCLC staging system **(D)**, serum AST **(E)**, MVI **(F)**, TNM stage **(G)**, serum ALP **(H)** and nomogram stage **(I)** of sorfenib cohort.

**Table 1** illustrates the results of the univariate analysis as well as multivariate analysis. Univariate analysis of OS exhibits that low levels of GPC3 (*P* < 0.001 **Figure 3A**), pERK (*P* < 0.001, **Figure 3B**), pAKT (*P* < 0.001, **Figure 3C**), serum AST (*P* = 0.004, **Figure 3E**) and serum ALP (*P* = 0.003, **Figure 3H**), BCLC stage 0/A (*P* < 0.001, **Figure 3D**), without MVI (*P* = 0.001, **Figure 3F**), TNM stage I/II (*P* = 0.002, **Figure 3G**) had a significant association with better OS in the sorafenib group. Multivariate analyses demonstrated that serum AST, BCLC staging system, GPC3, pERK, and pAKT were independent risk factors associated with OS. Diminished levels of GPC3, pERK, pAKT, serum AST, and BCLC stage 0/A predicted better OS of patients in the sorafenib category. At the same time, the low level of GPC3 (p < 0.001) and BCLC stage 0/A (p = 0.043) also associated with the better TTR for sorafenib use in HCC patients (**Figure S3**).

### Constructing and Validating the Prognostic Prediction Nomogram

**Figure 4:**
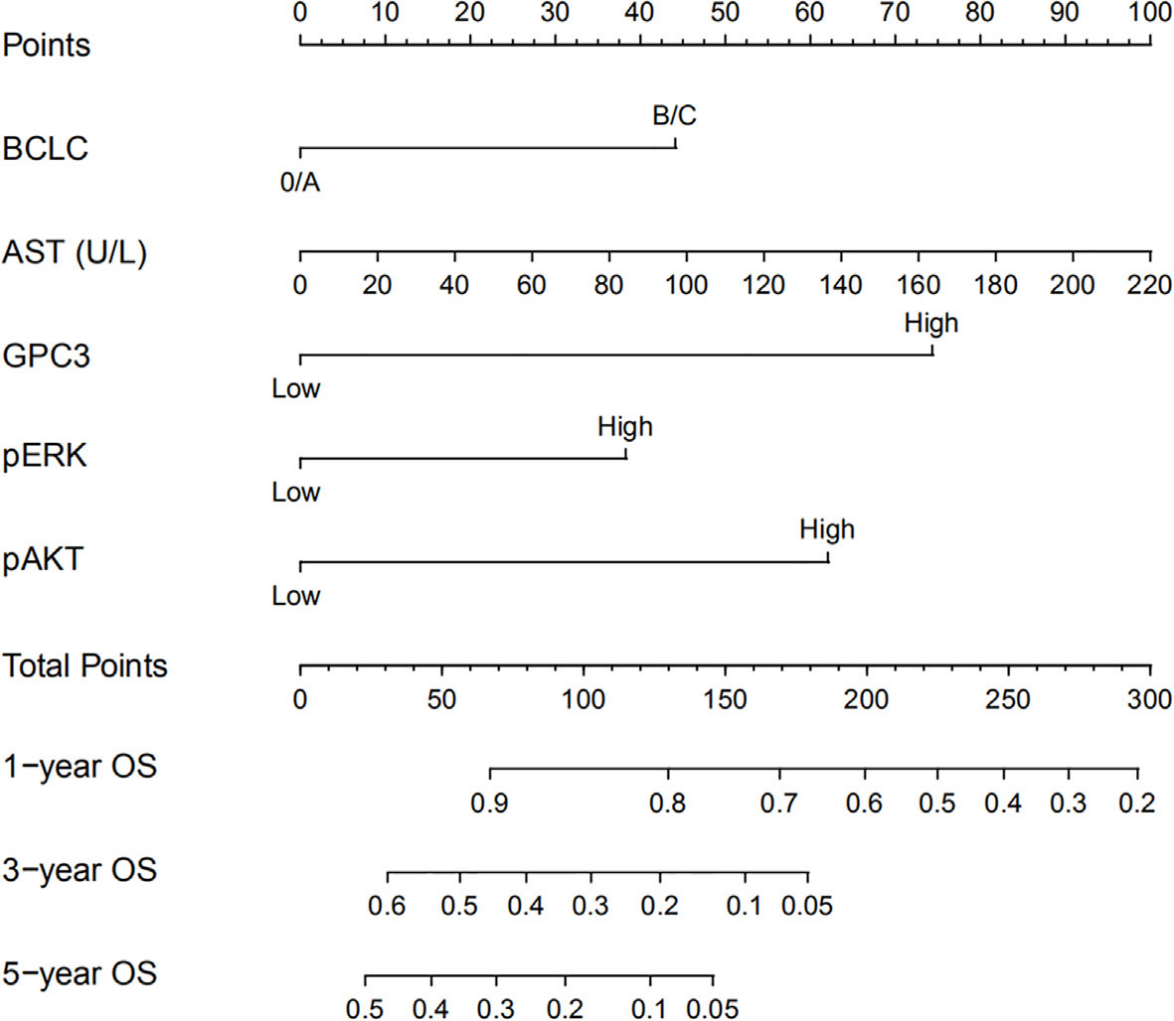
Nomogram for predicting OS of patients who received sorafenib after liver resection for Hepatitis B Virus related HCC. To use the nomogram, an individual patient’s value is located on each variable axis and a line is drawn upwards to determine the number of points received for each variable value. The sum of these numbers is located on the total points axis and a line is drawn downwards to the survival axes to determine the likelihood of 1-, 3- or 5-year survival rate.

**Figure 5:**
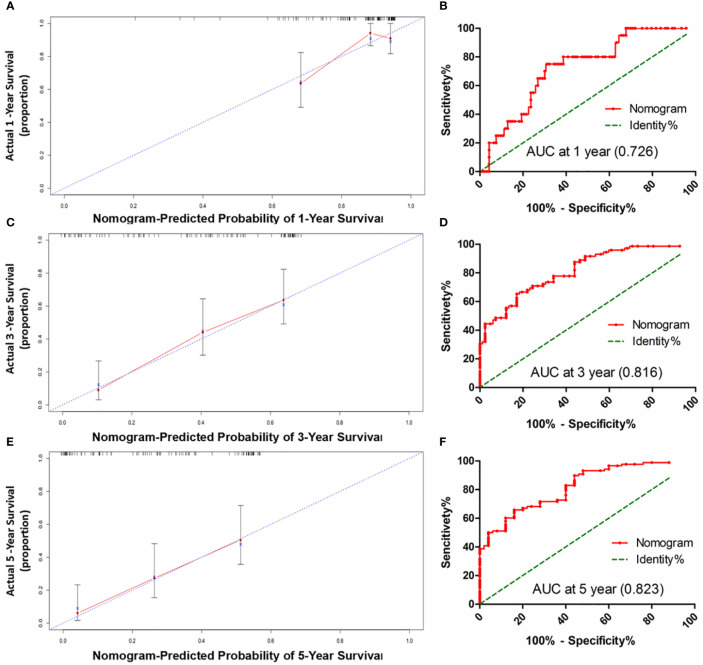
The calibration curve for predicting patients OS at 1-year **(A)**, 3-year** (C)** and 5-year **(E)** in the sorafenib cohort; The AUC values of ROC predicted 1-year **(B)**, 3-year **(D)** and 5-year **(F)**. OS rates of Nomogram in the sorafenib cohort.

HCC precision therapy is heavily reliant on the optimal combination of clinical variables and biomarkers to stratify patients (40, 41). Therefore, according to the independent prognostic factors identified by Cox regression, we further construct the prognostic prediction nomogram. The prognostic nomogram for the integration of all independent significant factors for OS in the sorafenib cohort can be seen in **Figure 4**. The nomogram was evaluated in terms of its discrimination power using ROC curves and calibration performance using C-index values. The 1-, 3-, and 5-year AUCs of the nomogram in the sorafenib cohort were 0.726, 0.816, and 0.823, respectively (**Figures 5B, D**, **F**). For OS prediction, the C-index was found to be 0.73; 95% CI ranging from 0.67 to 0.78. **Figures 5A, C, E** show that the calibration plot for 1-, 3-, or 5-year survival probability following surgery and reflect that the prediction of nomogram is in optimal agreement with real-time observations. Furthermore, the nomogram can exactly classify patients into three prognostic subcategories having respective scores of ≤28, 28–122, and >122. The respective 5-year rates of OS of the three subgroups were 46.9, 17.5, and 0% in the sorafenib cohort (*P* < 0.001, **Figure 3I**). The respective mean OS of nomogram stageI, stageII, and stage III in sorafenib cohort was found to be 56.0, 36.0, and 16.0 months. However, the respective OS of the three subgroups was 41.2, 29.5, and 24.5 months in the control cohort. The nomogram also showed the prognostic value in the control group (*p* < 0.001, **Figure S4F**). In comparison to the control group, the sorafenib group was found to have better OS in stageI (*p* < 0.001, **Figure S5A**) and stage II (*p* = 0.020, **Figure S5B**), but no significant difference was observed in stage III (*p* = 0.566, **Figure S5C**).

### Comparing the Accuracy of Prediction Between Nomogram and Single Independent Factor

The predictive potential of the nomogram for the prognosis of HCC patients with or without sorafenib therapy following partial hepatectomy was compared with independent factors. Among these independent risk factors, only GPC3 (**Figure S4A**) and BCLC system (**Figure S4D**) showed the prognostic value in the control cohort. The C-indices for OS prediction in the sorafenib cohort were 0.59 for BCLC staging system, 0.61 for serum AST, 0.62 for GPC3, 0.63 for pERK, 0.58 for pAKT, which were considerably less than the C-indices predicted by the nomogram (0.73; P < 0.001).

## Discussion

Some HCC patients initially respond to sorafenib; however, they succumbed to disease progression in later stages, thus limiting the benefits of sorafenib (42, 43). It is noteworthy that HCC is a highly heterogeneous malignancy in different individuals, thus it might show variable responses to sorafenib. Thus, this results in an increasing need for biomarkers regarding the selection of patients as well as prediction of response. Recently, the understanding of the underlying mechanism that influences the responses of HCC towards sorafenib has increased (41). Llovet et al. generated a newly 146-gene signature and was capable of recognizing 30% of patients who benefitted from sorafenib (4). A recent study reported that FLT3 might be able to predict sorafenib benefit in HCC patients. Numerous other works have reported that amplifying VEGFA, FGF3/FGF4, or FGF19 may potentially predict HCC response to sorafenib (44, 45). However, for sorafenib, no effective biomarkers of response have been identified (4). This work revolves around developing a nomogram to accurately make predictions regarding patient survival in HCC, for individuals exposed to sorafenib treatment after hepatic resection.

The prognostic significance of tumor cell serum GPC3 levels and GPC3 immunoreactivity in patients with HCC has been already established (46). EMT has been found to influence HCC resistance to sorafenib. Among various characteristics of EMT, an important hallmark is E-cadherin inhibition. Inhibition of E-cadherin leads to degeneration of the surrounding extracellular matrix due to the migration of primary malignant cells from their primary site and finally their migration into the blood vessels and eventual takeover of secondary organs (47). Wu et al. and Qi et al. showed that E-cadherin and GPC3 expression are correlated negatively in HepG2 cells (48, 49). Additionally, the level of E-cadherin was low in GPC3 overexpressing HCC tumor tissues (49). In GPC3-silenced HepG2 cells, a decrease in Slug and Snail and other EMT-related proteins and migration-related proteins (matrix metalloproteinase 2 and 9) was observed (48). In summary, these results indicate that EMT is promoted by GPC3 overexpression in HCC cells (16). The level of GPC3 was also showed in this nomogram. Our results showed that a low level of GPC3 in patients has a better OS than high in sorafenib cohort. The low level of GPC3 is significantly related to an improved OS inpatients subjected to sorafenib adjuvant therapy compared to those not treated with sorafenib. The low level of GPC3 was also associated with a better TTR in HCC patients treated with sorafenib after surgery.

pERK is a proxy for the sorafenib inhibition of the RAS/MAPK pathway *in vitro* in solid tumors (12). Several studies have proposed pERK as a candidate biomarker associated with prognosis following treatment with sorafenib, despite conflicting outcomes (50–52). The lack of a validated system of scoring for pERK immunostaining, and the variation among cohorts, endpoints, and detection techniques could be the possible reason behind these inconsistencies (4). The pERK level was included in the OS nomogram. In this study, the low level of pERK significantly correlated with an improved OS in patients exposed to sorafenib adjuvant therapy compared to those not treated with sorafenib. Decreased levels of pERK in patients led to better OS compared to those with high levels of pERK in the sorafenib cohort, but this was not found in the control group (**Figure S4**).

The pAKT level was included in the OS nomogram. Our results showed that sorafenib adjuvant therapy patients have better OS compared with patients not treated with sorafenib in a low level of pAKT cohort, and low levels of pAKT patients have better OS than high levels in sorafenib cohort. This result is supported by previous reports. Many studies have revealed that in sorafenib-resistant HCC cells, the Akt pathway is highly activated (53–56), and inhibition of Akt can potentially reverse this resistance by shifting autophagy from a role in cellular protection to a mechanism promoting death (53). Besides, the response towards sorafenib is impaired in HCC due to irregular p-AKT activation (57, 58). EMT has been observed to impact sorafenib resistance to HCC (57), and hyperactivity of PI3K/AKT signaling is a major originating reason (58, 59). In this trial, we arrived at the result that patients with low pAKT expression in the sorafenib cohort had a better prognosis, but for the control group, this trend was non-existent. Simultaneously, among the patients with low pAKT level, patients who received sorafenib therapy after surgery had a better OS than those who did not.

The serum AST and BCLC levels were also shown in this nomogram. Serum AST is included in many HCC prediction systems (26, 27). Our results showed that low serum AST has a better OS as compared to high serum AST in the sorafenib cohort, but this trend was not found in the control group (**Figure S4**). In patients with low serum AST, those treated with sorafenib after surgery had a better OS than those who were not treated with sorafenib. Previous studies have focused on the advanced stage of BCLC in HCC patients, and this study found that sorafenib adjuvant therapy after surgery in the initial stage of BCLC had a better OS in comparison to those not treated with sorafenib. At the same time, postoperative use of sorafenib in patients with BCLC 0/A predicted better TTR. This suggests that adjuvant therapy with sorafenib after surgery may benefit some patients with HCC.

Our proposed nomogram can thus efficiently predict the prognosis of HCC patients treated with sorafenib post-surgery quite accurately. The 1-, 3-, and 5-year AUCs of the nomogram in the sorafenib cohort were 0.726, 0.816, and 0.823, respectively. For OS prediction, the C-index was found to be 0.73; 95% CI ranging from 0.67 to 0.78. The prediction accuracy of the nomogram was better than that of a single independent factor. We also found that treatment with sorafenib after surgery in nomogram stage I patients had a significant benefit, while nomogram stage II patients had a partial benefit, but nomogram stage III patients had no significant benefit. Thus, our nomogram can be employed for predicting prognosis in patients with HCC exposed to sorafenib therapy after surgery, selecting appropriate candidates for potentially successful adjuvant therapy, and patient’s stratification in a randomized controlled trial design based on accurate prognostic stratification. At the same time, the model has the potential to facilitate active communication between patients and doctors about postoperative sequential treatment and prognostic analysis (60).

This study has several inevitable limitations. First, only a single Chinese institution was used for the establishment of a nomogram. Secondly, the patients in the cohort had a background of HBV infection. HCV infection patients were not included. Since HCV infection is an important factor or HCC cancerization, especially in Western countries, it is not clear whether this nomogram is suitable for patients with a Western background. Third, this study only has a primary cohort but no validation cohort, which is still a limitation and to a certain extent, might affect the results as well. Finally, whether or not the proposed nomogram applies to individual patients receiving another adjuvant therapy other than sorafenib remains to be ascertained.

## Conclusion

To conclude, our proposed nomogram can be used to choose appropriate candidates for potential and effective sorafenib adjuvant therapy after surgery. There is still an immense need for additional studies to establish whether or not it applies to other patient cohorts.

## Data Availability Statement

The original contributions presented in the study are included in the article/**Supplementary Material**; further inquiries can be directed to the corresponding authors.

## Ethics Statement

The research protocol was approved by the Ethics Committee of Shanghai Eastern Hepatobiliary Surgery Hospital. All the patients provided written informed consent.

## Author Contributions

WC, HD, and WD conceptualized and designed the study. WD, HY, and ZX performed the immunohistochemistry stain. WD, HY, YF, KY, YQZ, LH, YCZ, and ZC acquired and interpreted the data. All authors contributed to the article and approved the submitted version.

## Funding

This study was supported by grants from the National Natural Science Foundation of China (grant numbers: 81272662, 81472278, 81000969, 81502086 and 81472769). Funds for Creative Research Groups of National Natural Science Foundation of China (grant number: 81521091), Shanghai Municipal Commission of Health and Family Planning (grant number: 201840152), and National Commission of Health and Family Planning (grant number: 2018ZX10302207-004-005).

## Conflict of Interest

The authors declare that the research was conducted in the absence of any commercial or financial relationships that could be construed as a potential conflict of interest.

## Supplementary Material

Supplementary Figure 1 The diagrams with the flow of HCC patients.

Supplementary Figure 2 The indexes in predicting sorafenib benefit in patients stratified by prognostic factors. The OS of patients in overall cohort stratified by levels of GPC3 **(a)**, pERK **(b)**, pAKT **(c)**, Gender**(d)**, Age**(e)**, Serum AFP**(f)**, MVI**(g)**, BCLC stage**(h)**, TNM stage**(i)**, Child-pugh stage**(j)**, Serum ALT **(k)**, Serum AST **(l)**, Serum ALP **(m)**, Serum Cre **(n)** were compared between the sorafenib and control cohorts.

Supplementary Figure 3 Kaplan-Meier survival curves of TTR in the sorafenib cohort. GPC3 **(a)**, pERK **(b)**, pAKT **(c)**, BCLC staging system **(d)**, serum AST **(e)** and nomogram stage **(f)** of sorfenib cohort.

Supplementary Figure 4 Kaplan-Meier survival curves of OS in the control cohort. GPC3 **(a)**, pERK **(b)**, pAKT **(c)**, BCLC staging system **(d)**, serum AST **(e)** and nomogram stage **(f)** of control cohort.

Supplementary Figure 5 The nomogram stage in predicting sorafenib benefit in overall cohort. The OS were compared between the sorafenib and control cohorts of nomogram stage **(a)**, stage **(b)** and stage **(c)**.

## Abbreviations

ALT, Alanine aminotransferase; AST, Aspartate transaminase; AUC, The area under the ROC curve; C-index, Concordance index; FDA, Food and Drug Administration; GPC3, Glypican-3; HBcAb, Hepatitis B core antibody; HBsAg, Hepatitis B surface antigen; HBV, Hepatitis B virus; HCC, Hepatocellular carcinoma; HCV, Hepatitis C virus; HE, Hematoxylin-eosin; HGF, Hepatocyte growth factor; IFN, Interferon; IHC, Immunohistochemistry; MAPK/ERK, Mitogen-activated protein kinase/extracellular signal-regulated kinase; MRI, Magnetic resonance imaging; OS, Overall survival; pAKT, Phosphorylated AKT; pERK, Phosphorylated ERK; ROC, Receiver operating characteristic; TBIL, Total bilirubin; TNM, Tumor-node-metastasis classification system; TTR, Time to recurrence; VEGFR, Vascular endothelial growth factor receptors; WHO, World Health Organization.
