# Supplementary figures and images for: Prognostic Nomogram for Sorafenib Benefit in Hepatitis B Virus-Related Hepatocellular Carcinoma After Partial Hepatectomy
Source: Front Oncol. 2021 Feb 11;10:605057. doi: 10.3389/fonc.2020.605057 (PMC7906076; doi:10.3389/fonc.2020.605057)

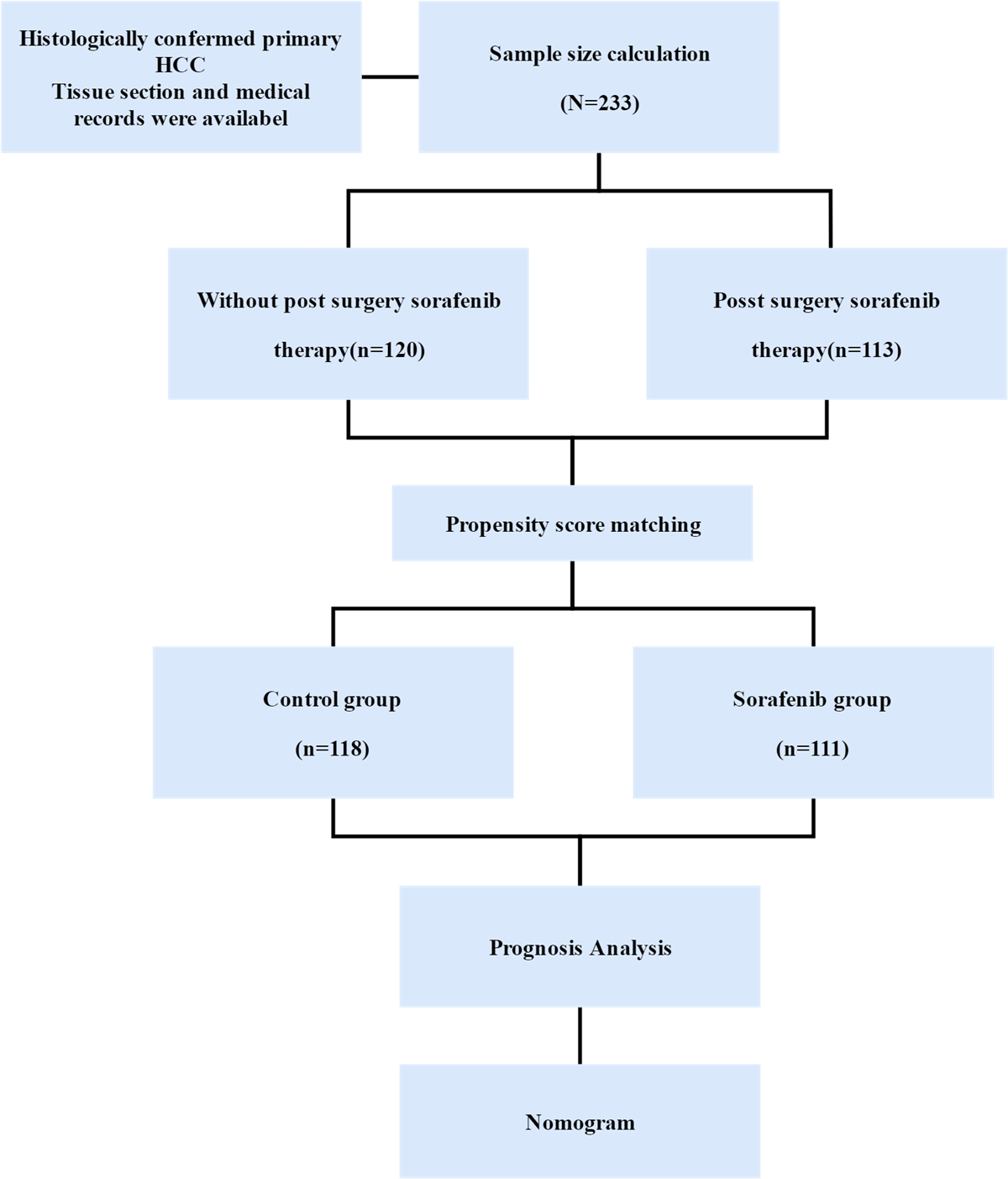

Supplement: Supplementary Figure 1 — The diagrams with the flow of HCC patients. [file Image_1.tif]

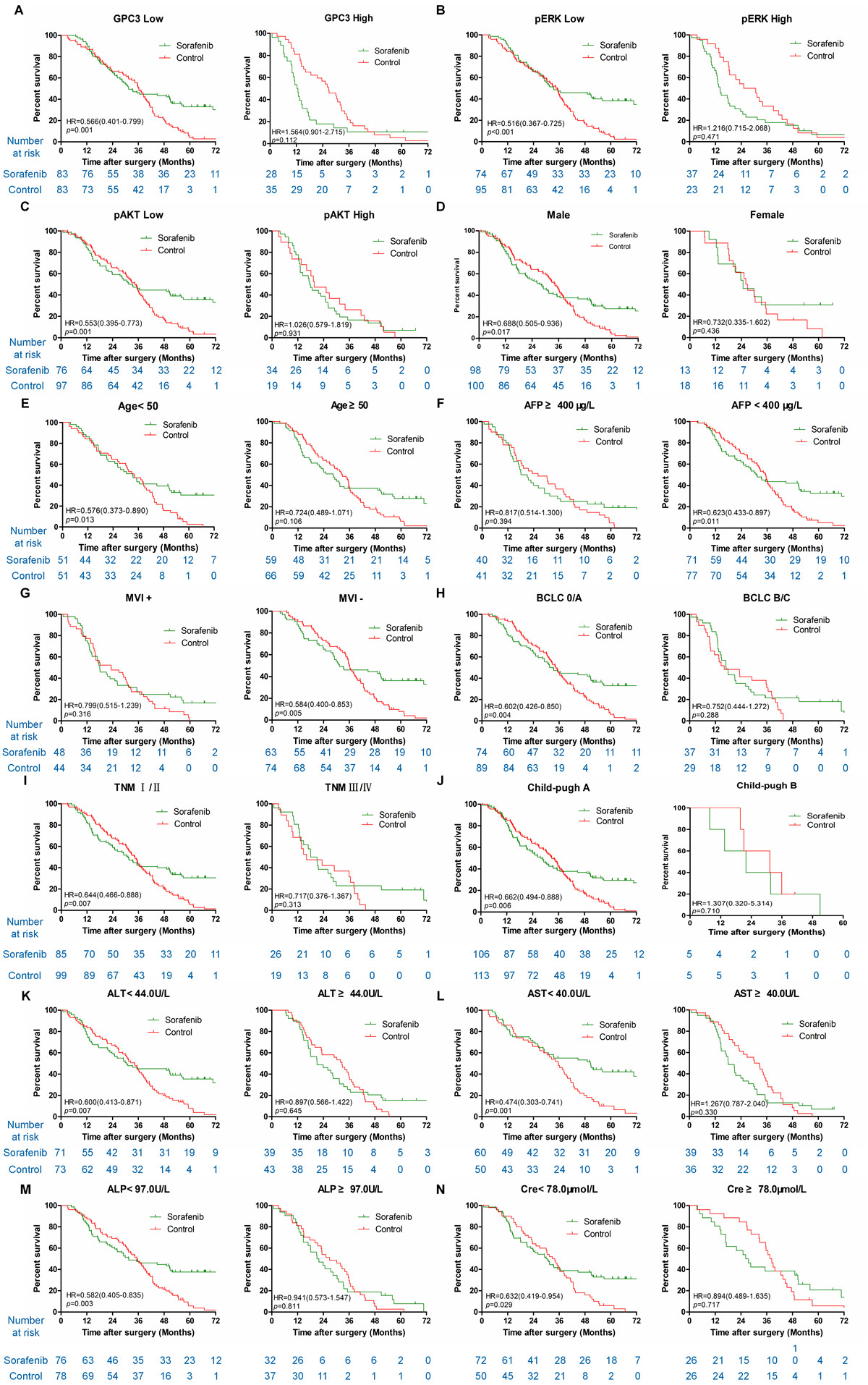

Supplement: Supplementary Figure 2 — The indexes in predicting sorafenib benefit in patients stratified by prognostic factors. The OS of patients in overall cohort stratified by levels of GPC3 (a), pERK (b), pAKT (c), Gender(d), Age(e), Serum AFP(f), MVI(g), BCLC stage(h), TNM stage(i), Child-pugh stage(j), Serum ALT (k), Serum AST (l), Serum ALP (m), Serum Cre (n) were compared between the sorafenib and control cohorts. [file Image_2.tif]

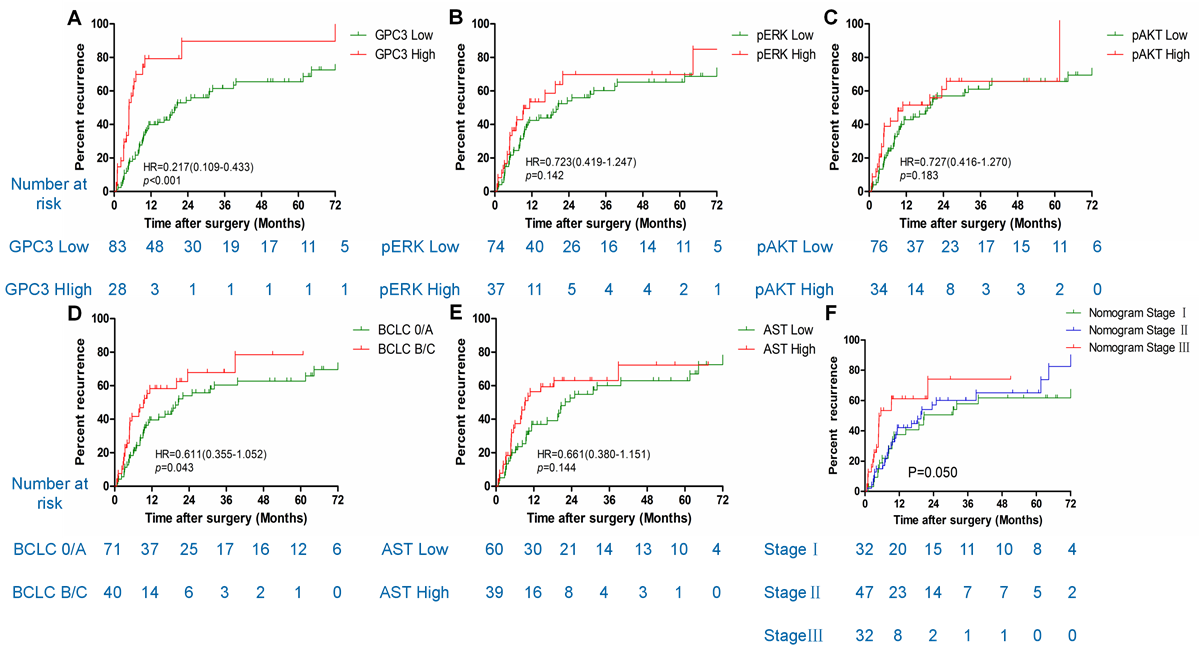

Supplement: Supplementary Figure 3 — Kaplan-Meier survival curves of TTR in the sorafenib cohort. GPC3 (a), pERK (b), pAKT (c), BCLC staging system (d), serum AST (e) and nomogram stage (f) of sorfenib cohort. [file Image_3.tif]

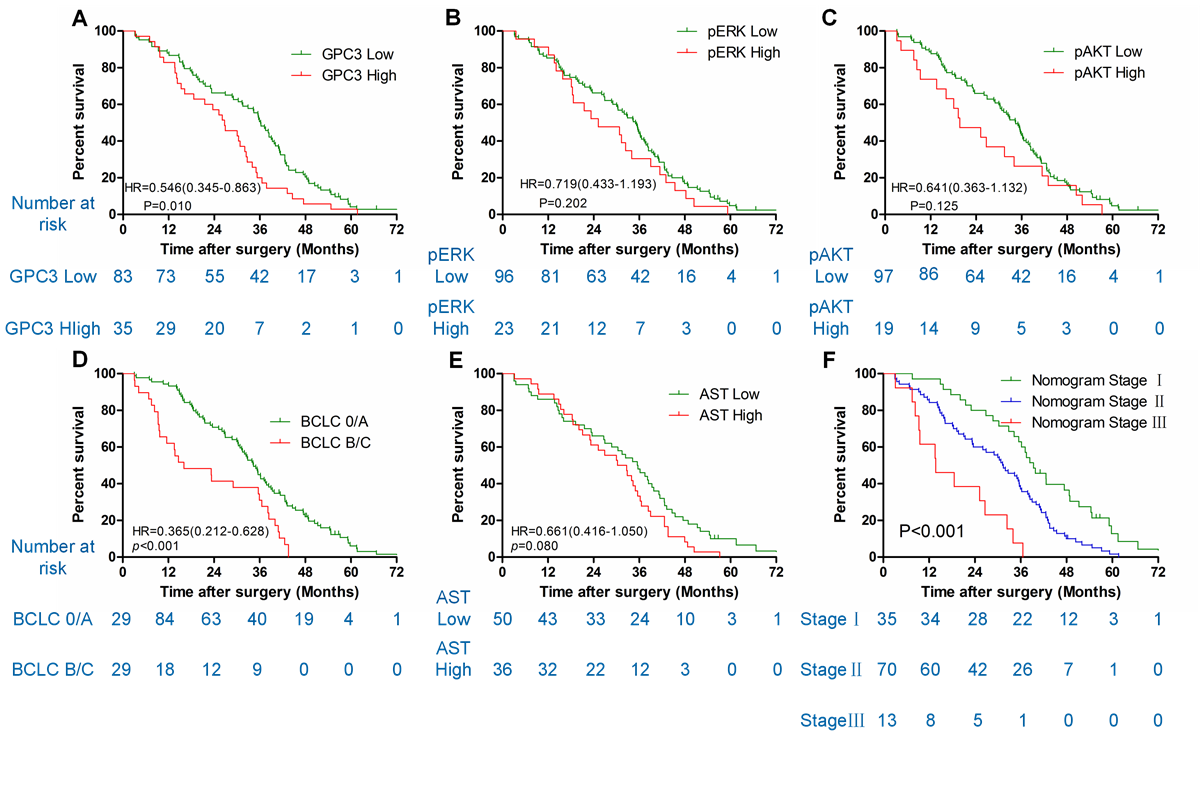

Supplement: Supplementary Figure 4 — Kaplan-Meier survival curves of OS in the control cohort. GPC3 (a), pERK (b), pAKT (c), BCLC staging system (d), serum AST (e) and nomogram stage (f) of control cohort. [file Image_4.tif]

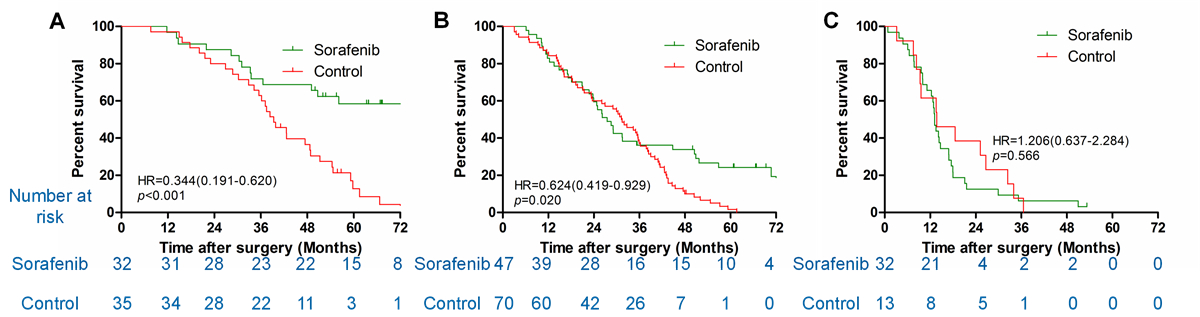

Supplement: Supplementary Figure 5 — The nomogram stage in predicting sorafenib benefit in overall cohort. The OS were compared between the sorafenib and control cohorts of nomogram stage (a), stage (b) and stage (c). [file Image_5.tif]
